# Erythropoietin, a putative neurotransmitter during hypoxia, is produced in RVLM neurons and activates them in neonatal Wistar rats

**DOI:** 10.1152/ajpregu.00455.2017

**Published:** 2018-02-14

**Authors:** Naoki Oshima, Hiroshi Onimaru, Akira Yamagata, Seigo Itoh, Hidehito Matsubara, Toshihiko Imakiire, Yasuhiro Nishida, Hiroo Kumagai

**Affiliations:** ^1^Department of Nephrology and Endocrinology, National Defense Medical College, Tokorozawa, Saitama, Japan; ^2^Department of Physiology, Showa University School of Medicine, Tokyo, Japan; ^3^Department of Physiology, National Defense Medical College, Tokorozawa, Saitama, Japan

**Keywords:** EPOR, erythropoietin, HIF-2α, hypoxia, RVLM neurons

## Abstract

Recent studies indicate that erythropoietin (EPO) is present in many areas of the brain and is active in the restoration of impaired neurons. In this study, we examined the presence of EPO and its role in bulbospinal neurons in the rostral ventrolateral medulla (RVLM). Hypoxia is often accompanied by a high blood pressure (BP). We hypothesized that EPO is produced in response to hypoxia in RVLM neurons and then activates them. To investigate whether RVLM neurons are sensitive to EPO, we examined the changes in the membrane potentials (MPs) of bulbospinal RVLM neurons using the whole cell patch-clamp technique during superfusion with EPO. A brainstem-spinal cord preparation was used for the experiments. EPO depolarized the RVLM neurons, and soluble erythropoietin receptor (SEPOR), an antagonist of EPO, hyperpolarized them. Furthermore, hypoxia-depolarized RVLM neurons were significantly hyperpolarized by SEPOR. In histological examinations, the EPO-depolarized RVLM neurons showed the presence of EPO receptor (EPOR). The RVLM neurons that possessed EPORs showed the presence of EPO and hypoxia-inducible factor (HIF)-2α. We also examined the levels of HIF-2α and EPO messenger RNA (mRNA) in the ventral sites of the medullas (containing RVLM areas) in response to hypoxia. The levels of HIF-2α and EPO mRNA in the hypoxia group were significantly greater than those in the control group. These results suggest that EPO is produced in response to hypoxia in RVLM neurons and causes a high BP via the stimulation of those neurons. EPO may be one of the neurotransmitters produced by RVLM neurons during hypoxia.

## INTRODUCTION

Erythropoietin (EPO) is a hematopoietic cytokine that is mainly produced in the kidneys and liver, particularly during hypoxia (11). Furthermore, EPO has been observed in other organs including the retina, muscle, gut, pancreas, gonads, uterus, and lung (5). Recently, the presence of EPO has also been shown in the brain (4). EPO in the brain is reportedly involved in the improvement of brain function after damage (1, 15, 22). EPO is regulated by hypoxia-inducible factor (HIF)-2 during hypoxia (6), and Chavez et al. (4) showed the expressions of HIF-2α and EPO in cultured cortical astrocytes. The presence of EPO in the brainstem has also been reported. Using brainstem-spinal cord preparations from mice, Soliz et al. (25) reported that EPO and EPOR are present in the mouse brainstem. They also showed that EPO controls ventilation and that an EPO antagonist suppressed the frequency of nerve activity at C_4_ (9).

Patients with hypoxia often exhibit high blood pressure (BP) and increased sympathetic nerve activity (8). Furthermore, chronic hypoxia reportedly induces a high BP (12), and a significant correlation in the oxygen partial pressure (Po_2_) is present between blood and cerebrospinal fluid (30).

From these reports, we hypothesized that the production of EPO increases in the brainstem in response to hypoxia and that this increase in EPO stimulates the activity of sympathetic-nerve-regulating neurons. Peripherally administered EPO passes through the blood-brain barrier (2), and an increase in BP has been observed in approximately half of EPO-treated patients (23, 24). These reports suggest that EPO causes a high BP by activating neurons that regulate sympathetic nerve activity.

The rostral ventrolateral medulla (RVLM) is known as a central region that regulates sympathetic nerve activities and controls BP (14). The RVLM neurons project to the intermediolateral cell column (IML), where they connect with the sympathetic preganglionic neurons (SPNs), and SPNs regulate the peripheral sympathetic function and BP (21). We thought that EPO was likely to be present in RVLM neurons and that it would affect the BP, especially under hypoxic conditions.

To the best of our knowledge, the direct effects of EPO on bulbospinal RVLM neurons have not been previously reported. Therefore, in this study, we examined the effects of EPO and soluble erythropoietin receptor (SEPOR, an endogenous EPO antagonist) (29) on bulbospinal RVLM neurons using brainstem-spinal cord preparations (19, 20). We also conducted a histological analysis to check for the presence of EPO, specific receptors for EPO, and HIF-2α on the RVLM neurons. Furthermore, we examined whether the levels of HIF-2α and EPO messenger RNA (mRNA) increased in response to hypoxia in RVLM areas.

## EXPERIMENTAL PROCEDURES

### General Preparations

Experiments were performed using brainstem-spinal cord preparations collected from 0- to 5-day-old Wistar rats, as previously described (19, 20). The experiment protocols were approved by the Institutional Review Board of the National Defense Medical College (Saitama, Japan) and were in accordance with the National Guidelines for the Conduct of Animal Experiments. The preparations were continuously superfused with a solution containing the following (in mmol/l): 124 NaCl, 5.0 KCl, 1.2 KH_2_PO_4_, 2.4 CaCl_2_, 1.3 MgCl_2_, 26 NaHCO_3_, and 30 glucose and were maintained at 25°C-26°C [artificial cerebrospinal fluid (aCSF)]. The pH (7.4) and oxygenation were maintained by bubbling 95% O_2_-5% CO_2_ through the solution.

### Patch-Clamp Electrodes

Electrodes were pulled in one stage from thin-wall borosilicate filament capillaries (GC100TF-10; outer diameter: 1.0 mm; Clark Electromed, Reading, UK) using a vertical puller. The electrodes had a tip diameter of 1.8–2.0 μm and a resistance of 4–6 MΩ. The electrode solution for the whole cell recordings consisted of the following (in mmol/l): 130 potassium gluconate, 10 HEPES, 10 EGTA, 1 CaCl_2,_ and 1 MgCl_2_, with the pH adjusted to 7.2–7.3 with KOH. The electrode tips were filled with 0.2% Lucifer yellow (Sigma, St. Louis, MO).

### Recording Procedure

A patch-clamp amplifier (AxoPatch, ID; Axon Instruments, Sunnyvale, CA) was used to record the membrane potentials (MPs). The RVLM neurons were sought from the ventral side of the medulla. To confirm that the recorded RVLM neuron was a bulbospinal neuron, the existence of antidromic action potentials in the RVLM neurons was examined by electrical stimulation of the IML at the Th_2_ level using a tungsten electrode (30-μm tip diameter; Unique Medical, Tokyo, Japan). All the data were recorded and analyzed using PowerLab (AD Instruments, Colorado Springs, CO). During the whole cell recordings, neurons were labeled with 0.2% Lucifer yellow (lithium salt; Sigma) by either spontaneous diffusion or iontophoresis.

### Experimental Protocols

#### Protocol 1.

During the recording of the MPs of the bulbospinal RVLM neurons, the preparations were superfused with EPO (0.1–2 nmol/l; KYOWA KIRIN) or SEPOR (3 nmol/l; Sigma) dissolved in aCSF. The duration of each drug superfusion was 3–10 min. The changes in the MPs were determined 3–5 min after the start of superfusion with each drug. The bulbospinal RVLM neurons were superfused with tetrodotoxin solution (TTX; 0.5 mmol/l (20); Sigma) for 10 min to block synaptic transmissions from other neurons to the recorded bulbospinal RVLM neurons. Thereafter, the neurons were superfused with EPO (0.5 nmol/l) dissolved in TTX solution and the MPs were recorded.

#### Protocol 2.

To examine whether SEPOR affects hypoxia-depolarized RVLM neurons, the hypoxia-depolarized RVLM was superfused with SEPOR (3 nmol/l). The hypoxic condition was made by bubbling 95% N_2_-5% CO_2_ through the solution.

#### Protocol 3.

To examine whether the changes in RVLM neuron activities caused by EPO or SEPOR superfusion were transmitted to the IML neurons, the MPs of the IML neurons were recorded and the RVLM areas were microsuperfused (10–30 μl) with EPO (1.5 nmol/l) or SEPOR (10 nmol/l) (19, 20).

### Immunofluorescence Staining

To determine the presence of specific transporters for these toxins histologically, immunofluorescence staining was performed. After the aforementioned experiments, the preparations were fixed for 1 h at 4°C in 4% paraformaldehyde in 0.1 M PBS, immersed in 18% sucrose-PBS overnight, embedded in optimal cutting temperature compound (Sakura Finetek, Japan), frozen on dry ice, and cut into 20-μm thick transverse sections, followed by immunofluorescence staining. The images were obtained using a conventional fluorescence microscope (LSM510; Carl Zeiss, Oberkochen, Germany).

### Protocols for Immunofluorescence Staining

The Lucifer yellow-stained RVLM neurons that responded to EPO were stained for EPOR. To examine the presence of EPO and HIF-2α in the RVLM, we performed immunofluorescence staining before EPO superfusion. The following primary antibodies (1:400 dilution) were used for immunofluorescence: goat anti-erythropoietin antibody (Funakoshi, Tokyo, Japan), mouse anti-HIF-2α antibody (Abcam), rabbit anti-EPOR antibody (Sigma), and rabbit anti-CD34 antibody (Abcam). To confirm that the examined area was a C1 area, the existence of tyrosine hydroxylase (TH)-positive neurons in the RVLM was also examined using mouse anti-TH antibody (Sigma). The secondary antibodies for fluorescence staining (1:1,000 dilution) were Alexa Fluor 546 goat anti-rabbit IgG (Molecular Probes/Invitrogen, Eugene, OR) Alexa Fluor 633 donkey anti-goat IgG (Molecular Probes/Invitrogen), and Alexa Fluor 633 goat anti-mouse IgG (Molecular Probes/Invitrogen).

### Real-Time RT-PCR

To confirm that EPO is produced during hypoxia in RVLM areas, we exposed RVLM areas to hypoxic conditions and measured the mRNAs of HIF-2α and EPO in these areas. Slice preparations were incubated in aCSF throughout the experiment.

#### Protocol 1.

In each experiment, two rats (0-day-old Wistar rats) were used (one for the control group and the other for the hypoxia group) (see Fig. 6*B*). From the medulla of each rat, 2 sections including the right or left RVLM region were prepared (see Fig. 6*A*). Throughout this experiment, the sections were incubated in aCSF in a 50-ml tube. The sections in each group were preincubated for 30 min. After preincubation, the sections in the hypoxia group were treated with the 5% CO_2_-95% N_2_ gas, while those in the control group were treated with 5% CO_2_-95% O_2_ gas, for 30 min. In both groups, the temperature was maintained at 32°C ± 1°C. After the 30**-**min treatment, the sections in both groups were oxygenated with 5% CO_2_-95% O_2_ for 3 h at room temperature (23°C ± 1°C) (see Fig. 6*B*) (26).

#### Protocol 2.

Extraction of the total RNA, reverse transcription into cDNA, and real-time PCR using the 7900HT Fast Real-Time PCR System (Applied Biosystems, Foster City, CA) were performed as previously described by Uchida et al. (28). We used the primer/probe sets of the TaqMan Gene Expression Assays for rat HIF-2α, EPO, and β-actin (Applied Biosystems). The relative amount of mRNA was calculated using the comparative C_t_ (ΔΔC_t_) method. All the amplification products were normalized against β-actin mRNA, which was amplified during the same reaction as an internal control.

### Statistics

The results are expressed as the means ± SE. Comparisons of the MPs recorded before and during (or after) superfusion with the drugs were performed using a paired *t*-test for paired observations. In the analysis of the levels of HIF-2α and EPO mRNA, the differences between the two experimental groups were assessed using the Student’s *t*-test. The statistical significance was set at *P* < 0.05. All the data were analyzed using JMP Pro 12 statistical software (SAS, Cary, NC).

## RESULTS

In total 80 bulbospinal RVLM neurons were examined. MP and the frequency of action potential (FAP) of those neurons under aCSF were as follows: MP, −45.4 ± 0.5 mV and FAP, 0.2 ± 0.1 Hz.

### Effects of EPO on Bulbospinal RVLM Neurons

**Fig. 1.**
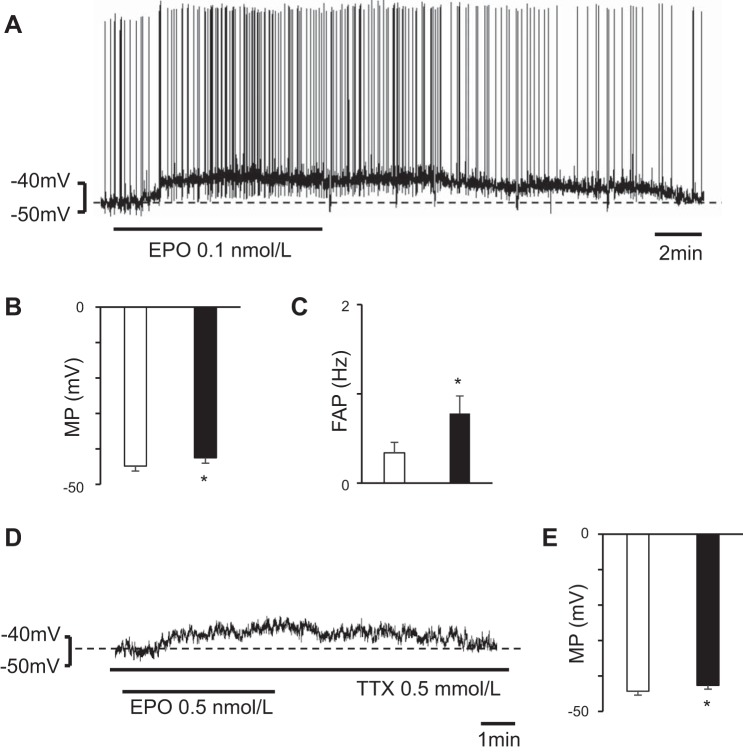
Bulbospinal rostral ventrolateral medulla (RVLM) neurons showing depolarization during erythropoietin (EPO) superfusion. *A*: neuron was depolarized by EPO superfusion (0.1 nmol/l). *B* and *C*: during EPO superfusion, the RVLM neurons were depolarized (*B*) and the frequency of action potential (FAP) in the RVLM neurons increased (*C*). Open bar, before EPO superfusion; solid bar, during EPO superfusion; values are means ± SE. *P < 0.05 vs. before EPO superfusion. *D*: neuron was depolarized during superfusion with EPO dissolved in a tetrodotoxin (TTX) solution. *E*: EPO dissolved in a TTX solution depolarized the RVLM neurons. Open bar, before superfusion with EPO dissolved in a TTX solution; solid bar, during superfusion with EPO dissolved in TTX solution; values are means ± SE. *P < 0.05 vs. before superfusion with EPO dissolved in a TTX solution.

To examine the effects of EPO on bulbospinal RVLM neurons, we superfused the bulbospinal RVLM neurons with EPO. EPO depolarized the RVLM neurons and increased FAP in those neurons at each of the examined concentrations (for 0.1 nmol/l, *n* = 11, the results are shown in Fig. 1, *A*–*C*) (for 0.5 nmol/l: before, −44.2 ± 0.6 mV; during, −42.0 ± 0.7 mV; *P* < 0.05; FAP: before, 0.3 ± 0.1 Hz; during, 0.5 ± 0.1 Hz; *P* < 0.05, *n* = 14) (for 2 nmol/l: before, −44.1 ± 0.8 mV; during, −41.5 ± 0.8 mV; *P* < 0.01; FAP: before, 0.1 ± 0.1 Hz; during, 0.5 ± 0.1 Hz; *P* < 0.01, *n* = 12). Furthermore, to examine whether EPO activated the RVLM neurons themselves, we superfused the RVLM neurons with EPO (0.5 nmol/l) dissolved in a TTX solution. The recorded bulbospinal RVLM neurons showed depolarization during superfusion with EPO dissolved in a TTX solution (*n* = 7) (Fig. 1, *D* and *E*). The results showed that EPO depolarizes the bulbospinal RVLM neurons themselves.

### Effects of SEPOR on RVLM Neurons

**Fig. 2.**
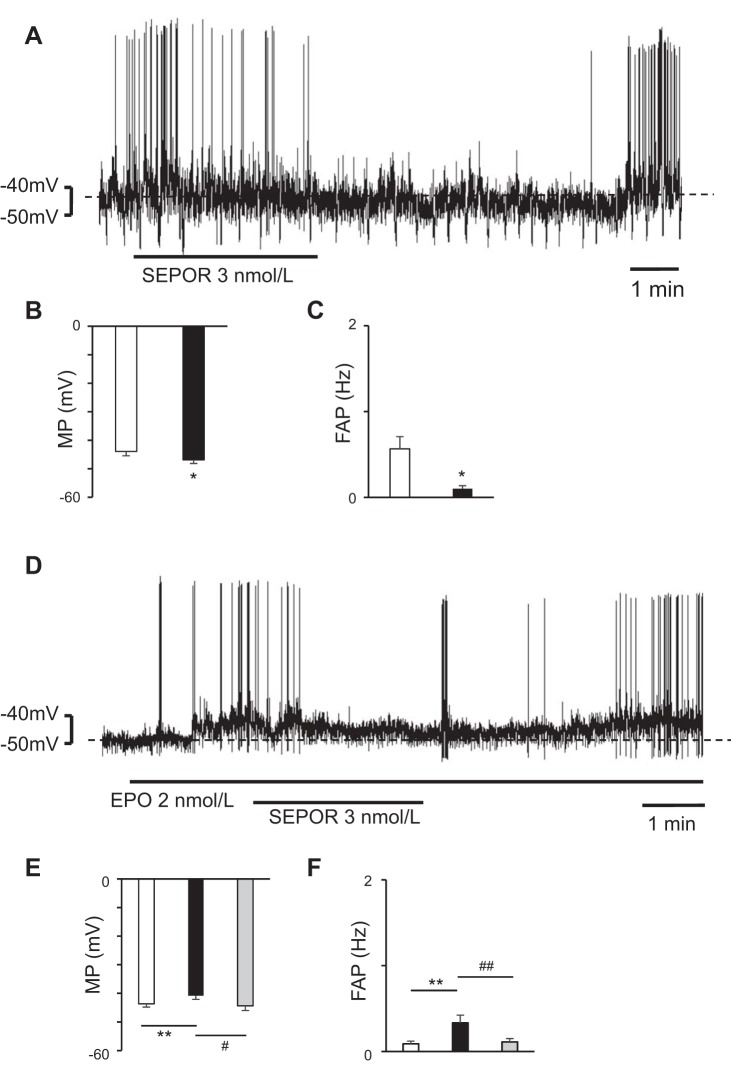
Bulbospinal rostral ventrolateral medulla (RVLM) neurons showing depolarization during soluble erythropoietin (EPO) receptor (SEPOR) superfusion. *A*: neuron was hyperpolarized by SEPOR. *B* and *C*: during SEPOR superfusion, the RVLM neurons were hyperpolarized (*B*) and the frequency of action potential (FAP) in the RVLM neurons decreased (*C*). Open bar, before SEPOR superfusion; solid bar, during SEPOR superfusion; values are means ± SE. *P < 0.05 vs. before SEPOR superfusion. *D*: neuron was depolarized by EPO and the addition of SEPOR hyperpolarized the EPO-depolarized RVLM neurons. *E* and *F*: during SEPOR superfusion, the EPO-depolarized RVLM neurons were hyperpolarized (*E*) and the FAP in the RVLM neurons decreased (*F*). Open bar, before EPO superfusion; solid bar, during EPO superfusion; gray bar, during superfusion with SEPOR dissolved in EPO solution; values are means ± SE. **P < 0.01 vs. before EPO superfusion. #*P* < 0.05 vs. during EPO superfusion. ##*P* < 0.01 vs. during EPO superfusion.

To examine whether EPO is present in the RVLM areas, we superfused the bulbospinal RVLM neurons with SEPOR (an endogenous EPO antagonist). SEPOR (3 nmol/l) hyperpolarized the bulbospinal RVLM neurons (*n* = 7) (Fig. 2, *A*–*C*). Furthermore, to examine whether SEPOR suppresses the effect of EPO on RVLM neurons, we added SEPOR (3 nmol/l) to EPO-depolarized bulbospinal RVLM neurons, and the addition of SEPOR to EPO (2 nmol/l)-depolarized RVLM neurons resulted in the hyperpolarization of the EPO-depolarized bulbospinal RVLM neurons (*n* = 9) (Fig. 2, *D*–*F*).

### Effects of SEPOR on Hypoxia-Depolarized RVLM Neurons

**Fig. 3.**
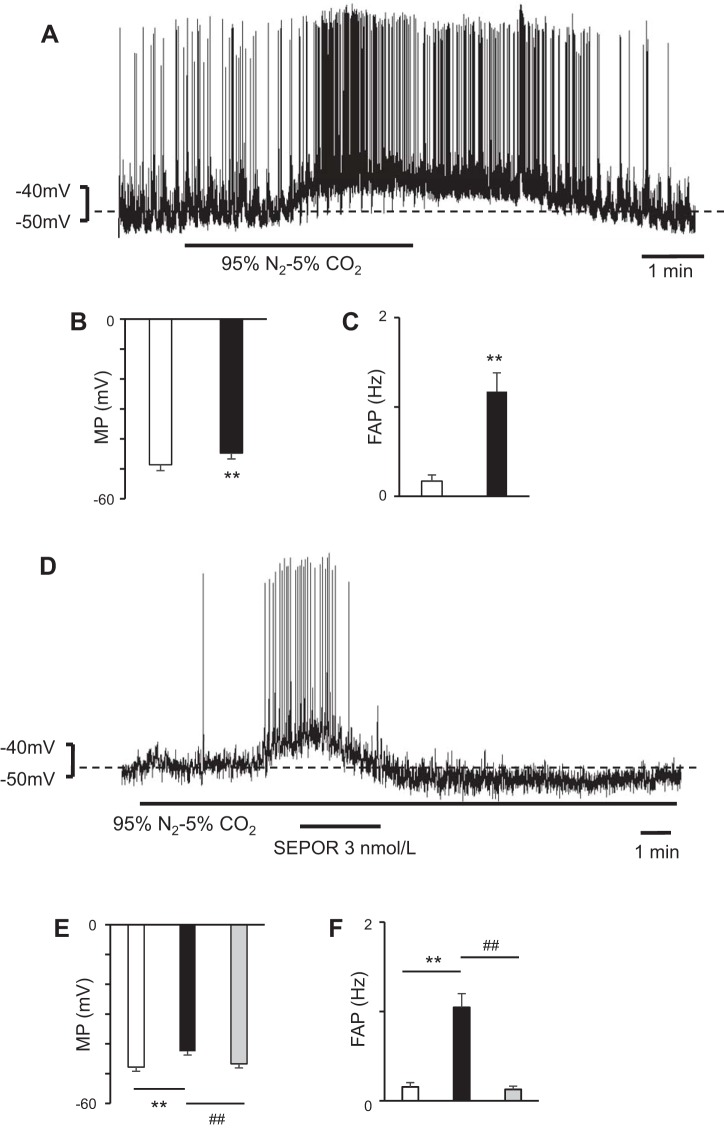
Hypoxia-depolarized bulbospinal rostral ventrolateral medulla (RVLM) neurons showing significant hyperpolarization during soluble erythropoietin (EPO) receptor (SEPOR) superfusion. *A*: neuron was depolarized during hypoxia. The hypoxia caused by bubbling 95% N_2_-5% CO_2_ depolarized the RVLM neuron. *B* and *C*: during hypoxia, the RVLM neurons were depolarized (*B*) and the frequency of action potential (FAP) in the RVLM neurons increased (*C*). Open bar, before hypoxia; solid bar, during hypoxia; values are means ± SE. **P < 0.01 vs. before hypoxia. *D*: addition of SEPOR hyperpolarized the hypoxia-depolarized RVLM neurons. In this case, the effect of SEPOR was very significant and prolonged. *E* and *F*: during SEPOR superfusion, the hypoxia-depolarized RVLM neurons were hyperpolarized (*E*) and the FAP in the RVLM neurons decreased (*F*). Open bar, before hypoxia; solid bar, during hypoxia; gray bar, during superfusion with SEPOR under hypoxic conditions; values are means ± SE. **P < 0.01 vs. before hypoxia. ##*P* < 0.01 vs. during hypoxia.

To examine the effect of hypoxia, we superfused RVLM neurons with aCSF bubbled with N_2_ 95%-CO_2_ 5%, and the RVLM neurons showed depolarization during hypoxia (*n* = 10) (Fig. 3, *A*–*C*). To examine the role of SEPOR on hypoxia-depolarized RVLM neurons, we added SEPOR to the solution. The addition of SEPOR hyperpolarized the hypoxia-depolarized RVLM neurons significantly (*n* = 10) (Fig. 3, *D*–*F*). These results suggest that EPO participates in the depolarization of RVLM neurons during hypoxia.

### Microsuperfusion With an EPO Solution Over an RVLM Area

**Fig. 4.**
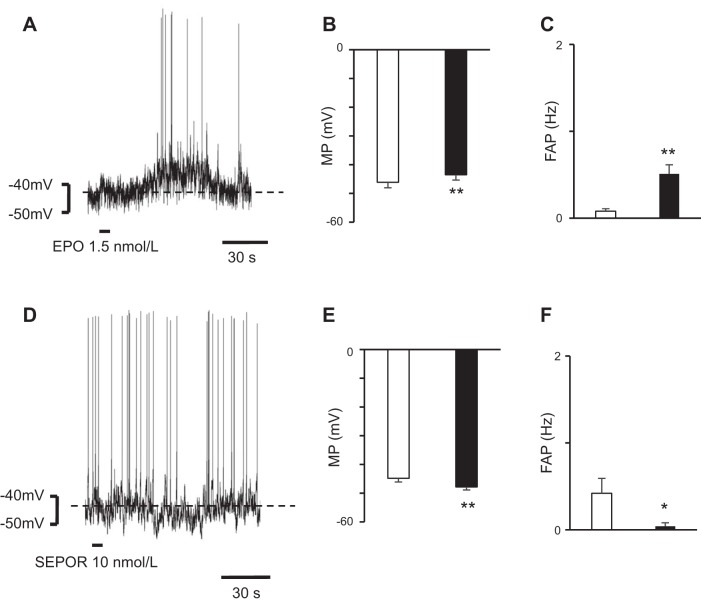
Change in membrane potentials (MPs) of intermediolateral cell column (IML) neurons after microsuperfusion with erythropoietin (EPO) or soluble erythropoietin receptor (SEPOR) over the rostral ventrolateral medulla (RVLM) area. *A*: IML neuron at the Th_2_ level was depolarized after EPO microsuperfusion over the RVLM area. *B* and *C*: after microsuperperfusion of the brain surface over the RVLM area with EPO, the IML neurons were depolarized (*B*) and the frequency of action potential (FAP) in the IML neurons increased (*C*). Open bar, before EPO superfusion; solid bar, after EPO superfusion; values are means ± SE. **P < 0.01 vs. before EPO superfusion. D: IML neuron at the Th_2_ level was hyperpolarized after SEPOR microsuperfusion over the RVLM area. *E* and *F*: after microsuperfusion of the brain surface over the RVLM area with SEPOR, the IML neurons were hyperpolarized (*E*) and the FAP in the IML neurons decreased (*F*). Open bar, before SEPOR superfusion; solid bar, after SEPOR superfusion; values are means ± SE. *P < 0.05, **P < 0.01 vs. before SEPOR superfusion.

In total 17 IML neurons were examined. MP and FAP of those neurons under aCSF were as follows: MP, −45.6 ± 1.3 mV and FAP, 0.2 ± 0.1 Hz. To examine whether the changes in the activities of the RVLM neurons induced by EPO were transmitted to the IML neurons, we microsuperfused the RVLM areas with an EPO solution (1.5 nmol/l) and observed the changes in the MPs of the IML neurons. Microsuperfusion of the brain surface over the RVLM with an EPO solution caused depolarization of the IML neurons at the Th_2_ level (*n* = 11) (Fig. 4, *A*–*C*). The latency, which is the period from the end of microsuperfusion over the RVLM areas to the beginning of the change in the MPs of the IML neurons, was 26.8 ± 4.8 s (Fig. 4*A*). Microsuperfusion of the brain surface over the RVLM with a SEPOR (10 nmol/l) solution hyperpolarized the IML neurons at the Th_2_ level (latency: 25.0 ± 3.2 s) (*n* = 6) (Fig. 4, *D*–*F*). These results show that the effects on RVLM neurons are transmitted to IML neurons.

### Immunoreactivity

**Fig. 5.**
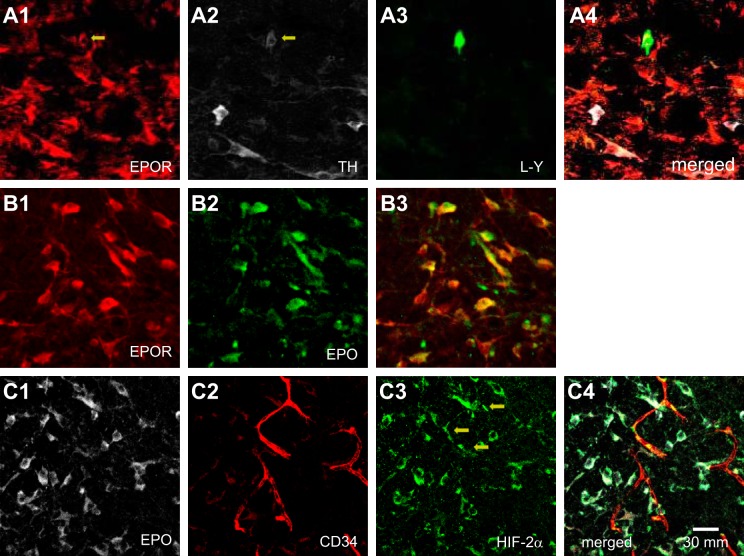
Histological examination of the bulbospinal rostral ventrolateral medulla (RVLM) neurons in a transverse section. *A*: erythropoietin (EPO)-depolarized bulbospinal RVLM neuron showed both EPOR and tyrosine hydroxylase (TH) immunoreactivities. *A1*: EPOR-immunoreactive neurons in the RVLM are stained in red. Arrow indicates the recorded bulbospinal RVLM neuron. *A2*: TH-immunoreactive neurons in the RVLM are stained in white. Arrow indicates the recorded neuron. *A3*: Lucifer yellow (L-Y)-stained bulbospinal RVLM neuron is stained in green. This neuron exhibited depolarization during EPO superfusion. *A4*: triple-merged image of *A1*, *A2*, and *A3*. *B*: EPOR-immunoreactive RVLM neuron exhibited EPO immunoreactivities. To confirm the presence of EPO in RVLM neurons, these examinations were performed before EPO superfusion. *B1*: EPOR-immunoreactive neurons. *B2*: EPO-immunoreactive neurons. *B3*: merged image of *B1* and *B2*. *C*: EPO-immunoreactive RVLM neuron exhibited hypoxia inducible factor (HIF)-2α immunoreactivities. These examinations were performed before EPO superfusion. *C1*: EPO-immunoreactive neurons. *C2*: CD34-immunoreactive vascular endothelial cells. *C3*: HIF-2α -immunoreactive cells. Arrows show vascular endothelial cells. *C4*: merged image of C1, *C2*, and *C3*.

To determine the presence of specific receptors for the EPO histologically, we performed immunofluorescence staining. Lucifer yellow staining was performed after the whole cell recordings of the bulbospinal RVLM neurons were completed (Fig. 5*A3*). Six RVLM neurons that showed depolarization during EPO superfusion were examined for EPOR immunoreactivity, and we confirmed that all of them were located in the RVLM and exhibited EPOR immunoreactivity (Fig. 5, *A1*, *A3*, and *A4*). Two of these six neurons also exhibited immunoreactivity for TH (Fig. 5, *A2*–*A4*).

Since the presence of EPOR was confirmed, we examined the presence of EPO in the RVLM area. To determine the presence of EPO histologically, immunoreactivity for EPO was examined in four rats. In the RVLM area, most of the EPOR-immunoreactive neurons also exhibited EPO immunoreactivity (Fig. 5, *B1*–*B3*).

Since EPO is produced through HIF-2α (Haase 2013), to determine the presence of HIF-2α histologically, we examined the immunoreactivity for EPO and HIF-2α. Most of the EPO-immunoreactive neurons also exhibited HIF-2α immunoreactivity (Fig. 5, *C1*, *C3*, and *C4*; examined in 3 rats). Since HIF-2α is sensitive to hypoxia, we examined whether HIF-2α-positive RVLM neurons were located near blood vessels. To confirm the presence of blood vessels, we examined the immunoreactivity for CD34. Not many HIF-2α-immunoreactive RVLM neurons were located near the blood vessels (Fig. 5, *C2*–*C4*), but some HIF-2α-immunoreactive cells (Fig. 5*C3*) were merged with the endothelial marker CD34 (Fig. 5, *C2*–*C4*).

### Real-Time RT-PCR

**Fig. 6.**
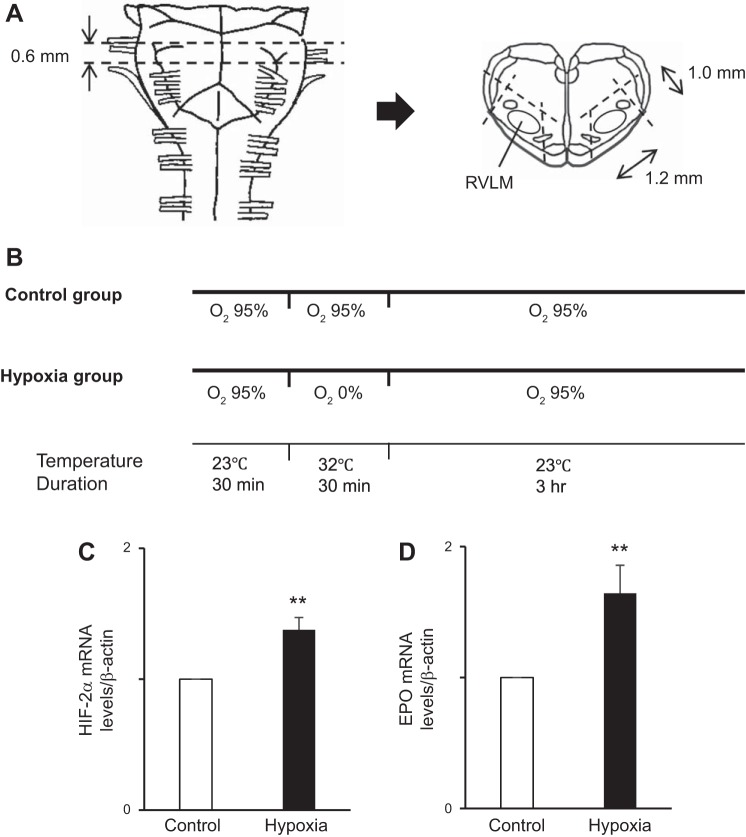
*A*: schema showing the medulla oblongata of the rat. From each rat, 2 sections containing rostral ventrolateral medulla (RVLM) regions were obtained by cutting along the dashed lines. *B*: experiment protocol. Two sections obtained from one rat were incubated under hypoxia or control conditions. The sections in the hypoxia group were exposed to the hypoxic conditions for 30 min. *C* and *D*: hypoxia inducible factor (HIF)-2α mRNA and erythropoietin (EPO) transcripts from the sections obtained from the ventral side of the medulla were quantitated relative to β-actin using real-time RT-PCR as described in experimental procedures. *C*: the level of HIF-2α mRNA in the hypoxia group was significantly higher than that in the control group. *D*: level of EPO mRNA in the hypoxia group was significantly higher than that in the control group. Open bar, control group; solid bar, hypoxia group. Data are normalized to β-actin expression and represent means ± SE of at least 2 samples per assay compared with the control group slices, the values of which were normalized to 1. **P < 0.01 vs. control.

In this experiment, we used seven pairs of rats (14 rats in total). The levels of HIF-2α and EPO mRNA at ventral sites of the medulla (containing the RVLM areas) were significantly higher in the hypoxic group than in the control group (*n* = 7) (Fig. 6, *C* and *D*). These results suggest that EPO is produced more under hypoxic conditions than under oxygenated conditions at ventral sites of the medulla.

## DISCUSSION

In this study, we examined whether EPO affects the electrophysiological properties of bulbospinal RVLM neurons and showed that EPO depolarized these neurons (Fig. 1, *A*–*C*). Furthermore, since the RVLM neurons showed depolarization during superfusion with EPO dissolved in a TTX solution (Fig. 1, *D* and *E*), we thought that EPO activated the bulbospinal RVLM neurons themselves. SEPOR is a competitor of EPOR. Since EPO that has bound to SEPORs cannot bind to EPORs, SEPOR is thought to inhibit the effect of EPO (29). In this study, since SEPOR hyperpolarized the bulbospinal RVLM neurons (Fig. 2, *A*–*C*), EPO was thought to be present in the RVLM and to have stimulated the RVLM neurons.

In this study, the bulbospinal RVLM neurons were significantly depolarized in response to hypoxia (Fig. 3, *A*–*C*). Nolan and Waldrop (17) showed that VLM neurons of rats were depolarized during hypoxia, and Mazza et al. (16) showed that cultured RVLM neurons of rats were depolarized during hypoxia induced by sodium cyanide (NaCN). Our results are compatible with these previous reports. Since EPO is produced more abundantly during hypoxia in the kidney, we hypothesized that EPO would be released in greater amounts during hypoxia than under conditions with a sufficient oxygen concentration in the RVLM and that it would stimulate the RVLM neurons more strongly. In this study, SEPOR significantly hyperpolarized hypoxia-depolarized bulbospinal RVLM neurons (Fig. 3, *D*–*F*), possibly supporting our hypothesis.

To confirm whether the changed activities of the RVLM neurons affected the IML neurons, we performed microsuperfusion with EPO or SEPOR in an RVLM area during whole cell recordings of an IML neuron at the Th_2_ level. Microsuperfusion with EPO in an RVLM area activated the IML neurons, while SEPOR in an RVLM area suppressed the activities of the IML neurons (Fig. 4, *A* and *B*). IML neurons projected by bulbospinal RVLM neurons are the final common pathway of the central nervous system and regulate peripheral sympathetic function and BP (3, 18). Therefore, the results of our study suggest that EPO administered to RVLM neurons increases the peripheral sympathetic nerve activity and probably raises the BP in vivo.

Since the specific receptor for EPO is EPOR (13), we examined the presence of EPORs on RVLM neurons. In our study, all the EPO-depolarized RVLM neurons showed immunoreactivity for EPOR (Fig. 5, *A1*–*A4*). These results suggest that EPO activates the bulbospinal RVLM neurons via EPORs expressed on these neurons. Because RVLM neurons include C1-catecholaminergic neurons (14, 19, 20), we examined the TH immunoreactivity of each EPO-depolarized RVLM neuron. Some of these neurons actually showed TH immunoreactivity (Fig. 5, *A1*–*A4*), suggesting that catecholaminergic RVLM neurons have EPORs.

Since SEPOR hyperpolarized RVLM neurons, we examined the presence of EPO in the RVLM. Most of the RVLM neurons that possessed EPORs showed EPO. In the kidney, HIF-2α is the principal regulator of EPO transcription, is mainly present in EPO-producing cells, and is activated under hypoxic conditions (10). Therefore, we also examined the presence of HIF-2α. As a result, most of the EPO-positive RVLM neurons exhibited HIF-2α (Fig. 5, *C1*, *C2*, and *C4*). These results suggest that HIF-2α mediates the transcriptional activation of EPO expression in RVLM neurons. Furthermore, some HIF-2α-positive cells were merged with CD34-positive cells (Fig. 5, *C2*–*C4*), suggesting the presence of HIF-2α in vascular endothelial cells. These results are supported by those of a previous report, which showed that HIF-2α was present in vascular endothelial cells (7).

The levels of HIF-2α and EPO mRNA at ventral sites of the medulla (containing the RVLM areas) were higher in the hypoxia group than in the control group. These results show that EPO is produced in the ventral sites of the medulla and that its production is stimulated during hypoxia.

To examine the hypoxic effect on RVLM neurons, we used a brainstem slice preparation (Fig. 6*A*) and compared the levels of HIF-2α and EPO mRNA in the hypoxia group with those in the control group. In this experiment, we referred to a method used in previous reports. To create an ischemic condition, Tamura et al. (26) exposed adult rat corticostriatal brain slices to 30 min of hypoxia (N_2_ 95%-CO_2_ 5%) with temporary oxygen/glucose deprivation. Then, after 1 h of oxygenated (95% O_2_-5% CO_2_) incubation with aCSF, they found that the protein expression of light chain 3-II had increased more in the ischemia group than in the control group. They also showed that 6 h of incubation after oxygen/glucose deprivation was required to detect obvious cell death in adult brain slice preparations. To examine the effect of hypoxia on EPO mRNA, we used slice preparations obtained from 0-day-old rats and incubated them with aCSF. We considered that the inside of the sections would be perfused more easily in neonatal rats than in adult rats because the neurons are not supported strongly by other surrounding tissues, such as astrocytes and fibers. Therefore, slice preparations obtained from 0-day-old rats were thought to be suitable for examining a few hours of cellular activity. A previous report showed that EPO mRNA significantly increases after 3 h of hypoxia in the mouse brain (26). Based on these studies (26, 27), we examined the level of EPO mRNA at 3 h after 30 min of hypoxia.

In conclusion, EPO was present in the RVLM neurons and increased the activities of bulbospinal RVLM neurons directly via the EPORs. The presence of HIF-2α in the RVLM neurons was shown histologically. Furthermore, during hypoxia, the production of EPO mRNA increased at medullary ventrolateral sites (including RVLM areas). Based on these results, EPO is produced in response to hypoxia in RVLM neurons and activates these neurons, increasing sympathetic nerve activity. This mechanism may lead to hypertension in vivo. EPO may be a neurotransmitter of RVLM neurons during hypoxia.

### Perspectives and Significance

The mechanisms of hypertension due to hypoxia are not well known. Since EPO, which is thought to be produced more during hypoxia, has been shown in the brain (4), we examined the presence of EPO and its role in RVLM areas. EPO stimulated the bulbospinal RVLM neurons and the production of EPO mRNA increased more at medullary ventrolateral sites (including RVLM areas) during hypoxia. These results explain some of the mechanisms of hypertension induced by hypoxia. Hypertension due to increased sympathetic nerve activity has been shown in patients with hypoxia (8), and we think that EPO in the RVLM neurons plays an important role in these patients.

## DISCLOSURES

No conflicts of interest, financial or otherwise, are declared by the authors.

## AUTHOR CONTRIBUTIONS

N.O., H.O., Y.N., and H.K. conceived and designed research; N.O. performed experiments; N.O., A.Y., S.I., H.M., T.I., and H.K. analyzed data; N.O. and H.K. interpreted results of experiments; N.O. prepared figures; N.O. drafted manuscript; N.O. and H.K. edited and revised manuscript; N.O. and H.K. approved final version of manuscript.
